# General Commentary to the “Management of Biochemical Recurrence after Primary Localized Therapy for Prostate Cancer” by Darwish O. M. and Raj G. V.

**DOI:** 10.3389/fonc.2012.00126

**Published:** 2012-09-27

**Authors:** Mark Shilkrut, Felix Feng, Daniel A. Hamstra

**Affiliations:** ^1^Department of Radiation Oncology, University of Michigan Health SystemAnn Arbor, MI, USA

TO THE EDITOR: In a recently published review on management of patients with biochemical failure (BF) following primary definitive therapy for localized prostate cancer (PCa), Darwish and Raj (2012) stated that following radical prostatectomy (RP) patients who are at high risk for recurrence after radiation therapy (RT), based upon a salvage nomogram, should be spared from this treatment modality justifying it by a range of toxicities it causes. Additionally, in the algorithm for management of BF after primary therapy they suggested that patients with a short prostate-specific antigen (PSA) doubling time (PSA-DT) and PSA > 10 ng/mL should not be offered salvage RT at all. Do these recommendations provide the optimal use of salvage RT following RP? Unfortunately, we feel that they do not.

While we agree with the authors that patients with higher PSA at the time of radiation (RT), short PSA-DT, high Gleason score, and other “bad” prognostic characteristics do generally worse than patients without those features, it does not mean that salvage therapy should categorically not be discussed and potentially offered to patients at high risk of recurrence. As a matter of fact, in the study that was cited in the manuscript (Stephenson et al., 2007), select patients with some “bad” prognostic features did remarkably well after salvage RT. The 6-year progression-free estimate in patients with PSA-DT ≤ 10 months and GS 8–10 with low PSA was 41%. When looking at PSA-DT, in particular those with the shortest doubling time may be at the greatest risk for PCa-specific mortality (PCSM). Therefore, although salvage RT may be less likely to prevent recurrence in this group in the ones who are salvaged with RT the benefit may be profound. This is supported by the analysis from Trock et al. (2008) who found, in a retrospective analysis of 635 patients with BF after RP, that there was an increase in PCa-specific survival after salvage RT in patients with short PSA-DT ≤ 6 months only and not in those with a longer PSA-DT. Ina similar retrospective analysis of 432 patients with BF after RP, the beneficial effect of salvage RT on all-cause survival was more prominent in patients with PSA-DT ≤ 6 months than in patients with PSA-DT > 6 months, with adjusted hazard ratios of 0.35 (95% CI 0.17–0.72, *p *= 0.042) and 0.6 (95% CI 0.37–0.98, *p *= 0.04), respectively (Cotter et al., 2011). All these data suggest that salvage RT is effective and local control is still important in many patients with short PSA-DT, and PSA-DT should not be used to select patients that will not receive RT. As a result the suggestion by Darwish and Raj to avoid salvage RT in these patients would potentially deprive those with the greatest relative benefit in regard to PCSM and overall survival from receiving this therapy.

Similarly, we disagree with the authors that “every salvage local therapy…for patients with BF is associated with a significant risk of complications…” Late complications after adjuvant or salvage RT are well documented from prospective randomized trials and from retrospective series. The rate of severe side-effects ranges from low (all grade 3–4, ≤5%; Bolla et al., 2005; Pearse et al., 2008) to very low (<1%; Feng et al., 2007; Wiegel et al., 2009) and should not preclude caretakers from offering this life-saving treatment to their patients. While every treatment decision should be based on patient’s preferences and informed consent, one should remember how little benefit from toxic antineoplastic therapies some patients will accept, when it is their only chance for cure. When considering adjuvant chemotherapy for early breast cancer (with less impressive HR for all-cause mortality (Early Breast Cancer Trialists’ Collaborative Group, 2005), and whose toxicities include death, life-threatening infections, neuropathy, cardiomyopathy, secondary cancers, infertility etc., a 1%-improvement in the chance of cure, or additional life expectancy of 6 months were found to be sufficient by 50% of women to decide that adjuvant chemotherapy was worthwhile (Duric and Stockler, 2001). For a man with a rising PSA after RP the use of salvage RT is the only potentially curative therapy, it can be delivered with a low risk of long-term toxicities, and should be discussed with patients as part of multidisciplinary management.

Further, we would support enrollment of these men on a number of key clinical trials now being evaluated in this setting which aim to further refine the impact of the timing of RT, the use, and duration of androgen deprivation therapy, and the use of pelvic RT in this critical patient population (RADICALS; RTOG-0534). However, we would not, as the authors suggest, recommend that these patients be recommended to go directly to systemic therapy due to a misconception about both the potential benefits and likely harms of salvage RT.
